# The diagnostic value of the stump impingement reflex sign for determining anterior cruciate ligament stump impingement as a cause of knee locking

**DOI:** 10.1186/1758-2555-4-29

**Published:** 2012-08-28

**Authors:** Michael R Carmont, Rob E Gilbert, Christopher Marquis, Omer Mei-Dan, Dai Rees

**Affiliations:** 1 Sports Injury Surgery, The Robert Jones and Agnes Hunt Hospital, Oswestry, Shropshire SY10 7AG, UK; 2 Department of Orthopedics, Division of Sports Medicine and Hip Preservation, University of Colorado, Denver, Colorado, USA

## Abstract

**Background:**

The stump impingement reflex is a subtle bounce to the knee thought to be caused by hamstring contraction when the knee is brought into extension and the torn ACL stump impinges between the distal femur and the tibial plateau. We have studied the diagnostic value of this sign.

**Findings:**

From Feb 2008-Feb 2009, we audited 30 patients who underwent urgent arthroscopy for acutely locked knees. The presence of the stump impingement reflex prior to surgery was compared with the intra-operative findings. The diagnostic values of the stump impingement sign were found to be: Sensitivity 58%, Specificity 81%, Positive predictive value 70%, Negative predictive value 72% and Accuracy 71%.

**Conclusions:**

We believe that the stump impingement reflex is a specific sign for ACL stump impingement as a cause of knee locking. We recommend close inspection for this sign when examining locked knees.

## Introduction

A knee is considered locked when it acutely loses full extension, during both active and passive movements and is held or “locked” in flexion. The causes of knee locking may be related to intra-articular pathology [1] i.e. a mechanical block (86%) [2] or due to an acute haemarthrosis and hamstring muscle spasm [3,4].

Both Noyes and Dehaven comment that a ruptured anterior cruciate ligament (ACL) is the commonest cause of locking (72%) and with bucket handle meniscal tears (62%, 15%) and loose bodies/chondral injuries (20%, 6%) may frequently occur in their respective series [5,6]. Partial ruptures to the ACL may result in locking, although this is uncommon [4,7,8]. Accurate clinical examination is essential as the presence of intra-articular impingement necessitates urgent arthroscopy.

The clinical examination of a painful sensitive locked knee can be difficult [5]. Lachmans test and the pivot shift are very sensitive [9] although these findings are may be less accurate in the presence of an acute effusion [10] and full extension may be considered necessary to perform a pivot shift test [11].

When examining an acutely injured knee full extension is compared by gently lifting both legs off the couch with hands placed beneath the patient’s heels to allow the knee to fall into natural hyperextension. A knee in which the ACL stump impinges between the femur and the tibia does not fully extend and may have a subtle flexion movement or bounce at the point extension is restricted (Additional file 1). We have termed this sign the Stump Impingement Reflex [12].

We aim to determine the diagnostic value of the stump impingement reflex sign in patients with acutely locked knees.

## Method

We undertook a prospective audit on all patients who presented with a locked knee following acute injury from February 2008 to February 2009. The hospital’s Audit Committee granted approval for the study. This study has received approval from the audit committee of the Robert Jones and Agnes Hunt Orthopaedic Hospital.

**Figure 1:**
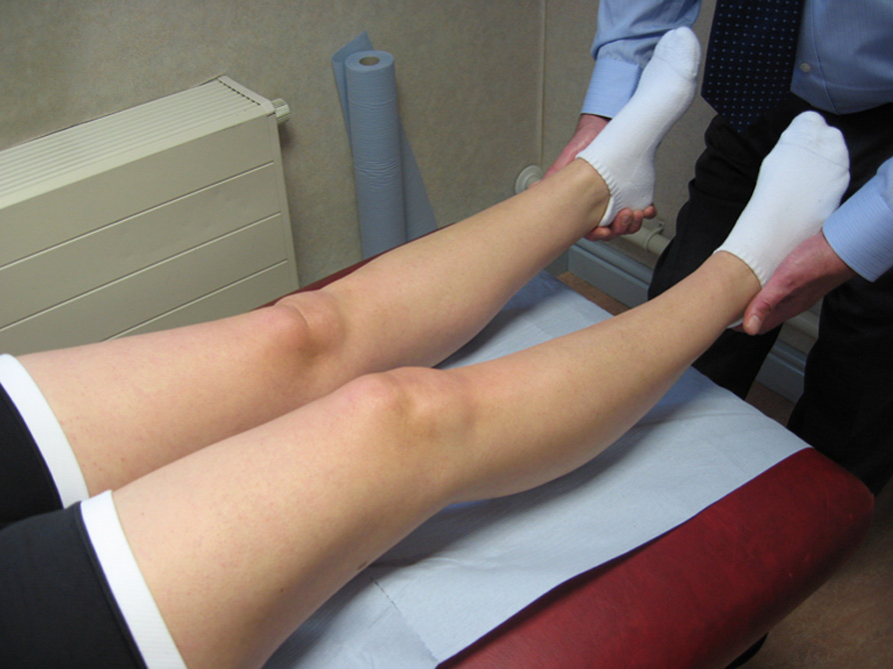
**Passive elevation of both legs off the examination couch, with the examiners hands beneath the patient’s heels allowing knee extension to be determined. **Knee locking is the acute inability for the knee to fully extend.

The patients included had all sustained an acute knee injury and subsequently had an acute inability to fully extend the knee both actively and passively. No threshold was set for the degree of fixed flexion deformity, the only criteria was that it had occurred acutely and was different to the non-injured side. The knee was then specifically assessed for extension noting the presence of the stump impingement reflex by placing a hand beneath each heel and gently lifting both legs simultaneously (Figure 1). The knee extends against gravity and is observed for range and pattern of movement looking particularly for the subtle flexion or “bounce” of the Stump Impingement Reflex (Test results). It is our routine practice for these patients to undergo urgent arthroscopy to permit full extension of the knee to be restored.

The arthroscopic findings were noted specifically looking for meniscal tears, loose bodies and ligament injury. The anterior cruciate ligament was assessed for the presence of injury and for stump impingment (Truth results). This allowed the diagnostic features of the test to be determined [13].

## Results

From Feb 2008-Feb 2009, 30 patients underwent urgent arthroscopy for acutely locked knees. The mean age was 25 years (16–56), 23 were male and 7 female, 16 patients had injured the left knee, 14 the right knee. At data collation 2 patients were noted to have previously undertaken ACL reconstruction and were excluded from data interpretation.

Pre-operatively 37% (10) patients demonstrated the stump impingement reflex (Figure 1), the remaining 20 did not. The mean fixed flexion deformity was 12 deg and flexion 120 deg.

**Figure 2:**
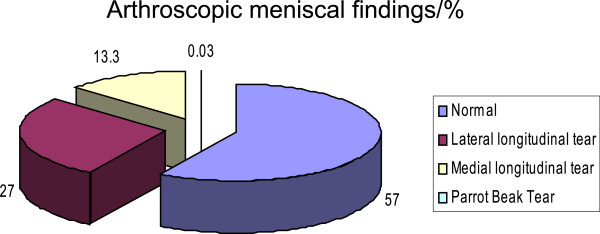
Arthroscopic meniscal findings/%.

At arthroscopy the ACL was had been injured in 63% (17) of cases: 11 completely torn with impingement, 2 partially torn with impingement, 3 strained but intact and in 1 case torn without impingement (Figure 2). The menisci were torn in 11 patients, 6 lateral and 5 medial, of these 10 had bucket handle tears and 1 parrot beak tear. 2 patients had co-existing bucket handle meniscal and ACL tears. Two patients had concurrent ACL and meniscal tears (Tables 1 &2).

**Table 1:** Arthoscopic diagnoses of the causes of knee locking

**Intra-articular pathology**	**Number**	**Percentage/%**
ACL complete rupture	9	30
ACL partial rupture	2	6.7
Meniscal tear Lateral	7	23
Meniscal tear Medial	3	10
ACL & Medial Meniscus	1	3.4
ACL & Lateral Meniscus	1	3.4
No structural pathology/Haemarthrosis/Pseudolocking	7	23
Total	30	

**Table 2:** Determination of the diagnostic validation of the stump impingement reflex sign

	**Scope (Truth) Positive**	**Scope (Truth) Negative**	
Exam (Test) Positive	7	3	10
Exam (Test) Negative	5	13	18
	12	16	28

Positive Predictive Value =7/10 = 0.7.

Negative Predictive Value =13/18 = 0.72.

Sensitivity =7/12 = 0.58.

Specificity =13/16 = 0.81.

Accuracy =20/28 = 0.71.

The diagnostic values of the stump impingement sign were found to be: Sensitivity 58%, Specificity 81%, Positive predictive value 70%, Negative predictive value 72% and Accuracy 71%.

## Discussion

Clinical examination of the acute knee aims to identify the diagnosis and also determine which knees are likely to benefit from urgent arthroscopy or focussed early physiotherapy. An acutely locked knee is uncomfortable, makes mobilisation difficult and physiotherapy painful. The recognition of the stump impingement reflex sign suggests a diagnosis of ACL impingement and so identifies those knees requiring prompt debridement. Stump debridement will allow the knee to settle and permit full extension improving outcome of ACL reconstruction [14] or permit physiotherapy rehabilitation. An acute haemarthrosis within the knee leads to the neurological inhibition of vastus medialis obliqus (VMO) resulting in weakness and atrophy [15,16]. In addition full knee extension is required to perform VMO exercises, optimising neuromuscular facilitation and promoting recovery [17].

The Stump Impingement Reflex Sign is a subtle one, detected by close inspection and appreciated by clinical experience or specific instruction. The sign is an objective test raising the possibility of inter and intra-observer error. To accumulate of an adequate number of acutely locked knees in one clinic would require a large patient population, a large clinical practice and prompt referral and so would be difficult. Also to delay surgery in these patients for error determination would be unethical. Repeated examination of an individual’s painful knee may well lead to guarding of the knee with quadriceps contraction further reducing the diagnostic value further. The mobility of the ACL stump also influences the reliability of this sign. The stump may return to its anatomical position at any time between and during individual extension examinations thereby not impinging.

Consistent with other series [5,6], the most common cause of knee locking was found to be a completely torn ACL (30%). Isolated lateral longitudinal bucket handle meniscal tears (23%) were more common than medial tears (10%) and isolated partially torn ACL injuries were least common (6.7%). Two patients had tears to the ACL in combination with bucket handle meniscal tears, one lateral meniscus, and one medial. Almost a quarter (23%) of patients had no evidence of meniscal injury and had either a normal ACL or a strained but intact ACL without impingement (a pseudo locked knee). Since Hilton’s initial work [18], the appreciation of the neurological innervation of individual intra-articular structures is only just being appreciated. Electromyographic observation of hamstring muscle activity following ACL stimulation has led to the recognition of an ACL-Hamstring reflex arc [19] whereas no reflex activity has been shown with meniscal stimulation [20]. The fact that only 1 out of 28 patients demonstrated the reflex under general anaesthesia suggests that the knee flexion response is centrally controlled possibly via pain receptors within the ACL stump [21].

ACL grafts have been shown to reform a hamstring reflex arc between 37–80 months following bone-patellar tendon-bone autograft ACL reconstruction [22]. Two patients had reinjured their knees at 42 and 96 months following reconstruction and if included, the specificity would increase to 83% and the negative predictive value to 75%.

We have determined the following diagnostic value of the stump impingement reflex sign: Sensitivity 58%, Specificity 81%, Positive predictive value 70%, Negative predictive value 72% and Accuracy 71%. We recommend close inspection for this sign when examining locked knees in the clinical setting. Identificaton of the reflex in the acutely painful locked knee may be of diagnostic value when other clinical signs may be less reliable.

## Consent

All patients gave their consent for inclusion in the study and data collection.

## Competing interests

The authors can confirm that there are no competing interests.

## Authors’ contributions

MC devised the study, analysed the data and wrote the manuscript. RG and CM collected the data. OMD assisted with writing the manuscript. DR provided the inspiration for and assisted with devising the study. All authors read and approved the final manuscript.

## Supplementary Material

Additional file 1 Video showing the subtle bounce of the knee caused by hamstring contraction in response to a stump of the ACL being stimulated by impingement within the nearside knee. Click here for file
